# Loss of miR-23b/27b/24-1 Cluster Impairs Glucose Tolerance via Glycolysis Pathway in Mice

**DOI:** 10.3390/ijms22020550

**Published:** 2021-01-07

**Authors:** Yong-Hui Jiang, Yuan-Yuan Man, Yue Liu, Chang-Jian Yin, Jia-Lin Li, Huang-Cong Shi, Han Zhao, Shi-Gang Zhao

**Affiliations:** 1 Center for Reproductive Medicine, Cheeloo College of Medicine, Shandong University, Jinan 250012, China; jiangyonghui126@126.com (Y.-H.J.); manyuanyuan_0018@163.com (Y.-Y.M.); syly130502@126.com (Y.L.); 17865195362@163.com (C.-J.Y.); jialinli1160@163.com (J.-L.L.); zjyyshc@126.com (H.-C.S.); hanzh80@yahoo.com (H.Z.); 2 Key Laboratory of Reproductive Endocrinology of Ministry of Education, Shandong University, Jinan 250012, China

**Keywords:** T2DM, impaired glucose tolerance, glycolysis, miR-23b/27b/24-1 cluster

## Abstract

Alterations in miRNAs are associated with many metabolic disorders, such as type 2 diabetes (T2DM). The miR-23b/27b/24-1 cluster contains miR-23b, miR-27b, and miR-24-1, which are located within 881 bp on chromosome 9. Studies examining the roles of miR-23b, miR-27b, and miR-24-1 have demonstrated their multifaceted functions in variable metabolic disorders. However, their joint roles in metabolism in vivo remain elusive. To investigate this subject, we constructed miR-23b/27b/24-1 cluster knockout (KO) mice. Compared with wild-type (WT) mice, the KO mice exhibited impaired glucose tolerance, which was accompanied by a reduction in the respiratory exchange rate (RER). These alterations were more noticeable after a high-fat diet (HFD) induction. Hepatic metabolomic results showed decreased expression of reduced nicotinamide adenine dinucleotide (NADH), nicotinamide adenine dinucleotide (NAD), phosphoenolpyruvic acid (PEP), and phosphoric acid, which are involved in the glycolysis pathway. The transcriptomic results indicated that genes involved in glycolysis showed a downregulation trend. qPCR and Western blot revealed that pyruvate kinase (PKLR), the key rate-limiting enzyme in glycolysis, was significantly reduced after the deletion of the miR-23b/27b/24-1 cluster. Together, these observations suggest that the miR-23b/27b/24-1 cluster is involved in the regulation of glucose homeostasis via the glycolysis pathway.

## 1. Introduction

Type 2 diabetes mellitus (T2DM) has increasingly grown to epidemic proportions over the last 30 years. The complications associated with this disease have a considerable impact on both the individual and the socioeconomic problems [1]. Patients with T2DM have an increased risk of cardiovascular disease (CVD), heart failure, and chronic kidney disease (CKD) [2]. As such, most research efforts have sought to elucidate its pathogenesis to increase the therapeutic options for diabetes mellitus. MicroRNAs (miRNAs) are involved in the pathogenesis of complex diseases, including T2DM [3,4].

The miR-23b/27b/24-1 cluster contains miR-23b, miR-27b, and miR-24-1, which are located within 881 bp on chromosome 9. All the three miRNAs in the plasma have been reported to be independently upmodulated upon type 1 diabetes progression [5]. MiR-23b plays important roles in cell growth and differentiation during development [6], cancer [7], cardiovascular disease [8], viral infections [9], and autoimmune diseases [10]. In addition, the miR-23b level was reduced in T2D patients with obesity [11]. Other results indicated that miR-23b and miR-27b were downregulated in muscle stem cells derived from T2DM patients [12]. Mechanistically, miR-27b can negatively regulate human adipocyte differentiation by targeting *LPL* or *PPAR-γ* genes [13,14]. Both miR-27b and miR-24 levels were decreased in the urine extracellular vesicles of patients with diabetic nephropathy [15]. The dysregulated pattern of miR-24 in patients with T2DM is controversial. Several groups have demonstrated that miR-24 was elevated in T2DM patients [16,17,18] and decreased after treatment with metformin [19]. Other studies have shown that the expression of miR-24 was reduced in T2DM patients [20,21,22]. Elevated expression of all three miRNAs has also been reported in the liver of high-fat diet (HFD) mice, and knockdown (KD) of miR-24 caused impaired hepatic lipid accumulation [23,24]. We also confirmed that these three miRNAs were all upregulated in ob/ob mouse livers (Figure S1). To the best of our knowledge, there are only two knockout studies on this cluster in mice related to bone marrow cellularity and angiogenesis [25,26]. However, the function of these three miRNAs together in glucose metabolism remains elusive.

Together, these observations led us to propose that the miR-23b/27b/24-1 cluster may be involved in the regulation of metabolic pathways. To test this hypothesis, we examined the effect of the ablation of the miR-23b/27b/24-1 cluster on glucose and lipid metabolism in mice under normal chow diet (NCD) or high-fat diet (HFD).

## 2. Results

### 2.1. Generation of miR-23b/27b/24-1 Cluster Knockout Mice

**Figure 1:**
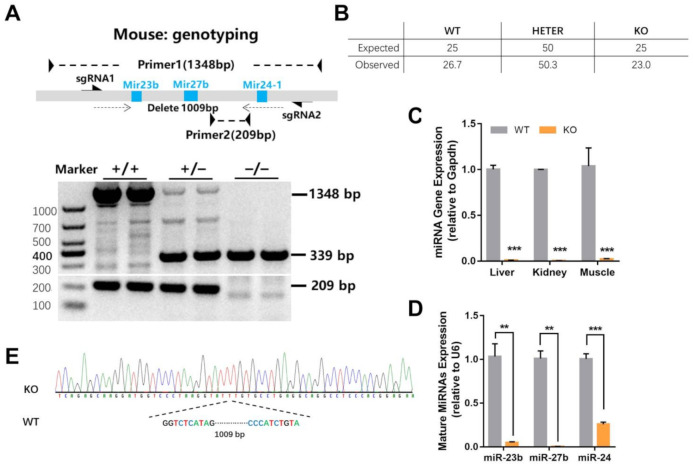
Generation of miR-23b/27b/24-1 cluster knockout mice. (**A**) The construction and genotyping of miR-23b/27b/24-1 cluster knockout mice. (**B**) The proportion of offspring genotypes obtained by mating heterozygous mice (*n* = 187). (**C**) The miRNA gene expression (relative to *Gapdh*) of liver, kidney, and muscle between wild-type (WT) and knockout (KO) mice (*n* = 3). (**D**) The expression of mature miRNAs expression (relative to *U6*) between the WT and KO mice (*n* = 4). (**E**) DNA sequencing results between WT and KO mice. Data were represented as mean ± SEM. Significance was determined by Student’s *t*-test analysis, ** *p* < 0.01, *** *p* < 0.001.

To better study the physiological effect of the miR-23b/27b/24-1 cluster, we constructed the whole miRNA cluster knockout (KO) mice (Figure 1A). The mice, obtained at Mendelian ratios, were viable and fertile with no gross abnormalities (Figure 1B). We also confirmed that the target region was effectively excised in KO mice. Real-time PCR assay of liver, kidney, and muscle indicated that the miR-23b-27b-24-1 cluster was almost undetectable in KO mice compared with that in WT mice (Figure 1C). Mature miR-23b, miR-27b, and miR-24-1 were also significantly reduced in the liver tissue of KO mice (Figure 1D). We next sequenced the genomic DNA, and the results revealed that the KO mice missed 1009 bases than the wild-type (WT) mice (Figure 1E).

### 2.2. Loss of miR-23b/27b/24-1 Cluster Impairs Glucose Tolerance

**Figure 2:**
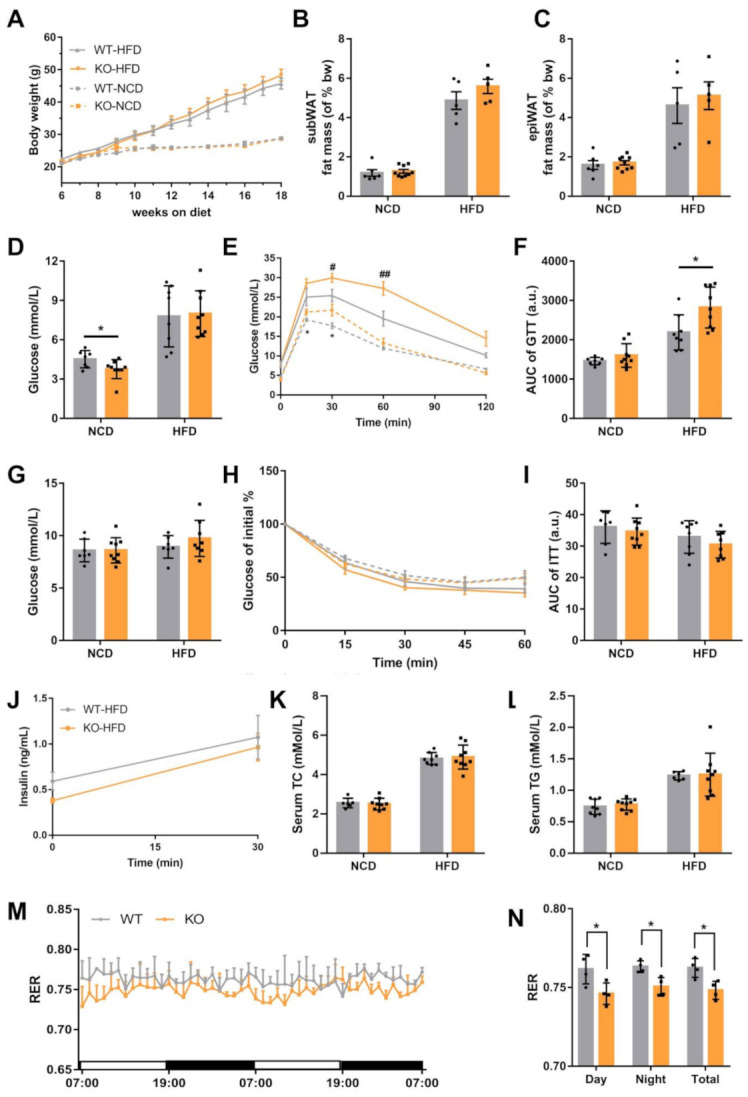
Loss of miR-23b/27b/24-1 cluster impairs glucose tolerance. (**A**) Body weight changes of WT and KO mice under normal chow diet (NCD) and high-fat diet (HFD) (*n* = 6–9). (**B**) Subcutaneous WAT (subWAT) and (**C**) epididymal WAT (epiWAT) fat mass of WT and KO mice (*n* = 5–9). (**D**) Fasting blood glucose levels after an overnight fast under different dietary conditions (*n* = 7–9). (**E**) Glucose tolerance test and area under the curve (**F**) in WT and KO mice under NCD or HFD (*n* = 7–9), * comparison of KO vs. WT under NCD, ^#^ comparison of KO vs. WT under HFD. (**G**) Random blood glucose levels measured under different dietary conditions (*n* = 7–9). (**H**) Insulin tolerance test and area under the curve (**I**) in WT and KO mice under NCD or HFD (*n* = 7–9). (**J**) Insulin levels after glucose stimulation under HFD (*n* = 8–9). (**K**) Levels of serum cholesterol and triglycerides (**L**) of WT and KO mice fed an NCD (*n* = 6–9) or HFD (*n* = 8–9). (**M**) Respiratory exchange ratio (RER) was measured during two successive days using metabolic cages. The daytime and nighttime RER averages are on the right panel (**N**). Mice were fed an HFD (*n* = 4). Data were represented as mean ± SD. Significance was determined by Student’s *t*-test analysis, * and ^#^ *p* < 0.05, ^##^ *p* < 0.01.

As HFD or ob/ob mice show elevated miRNA expression, we analyzed the metabolism indices under NCD and HFD conditions to better investigate the role of the miR-23b/27b/24-1 cluster. Overall, there was no significant difference in body weight between WT and KO mice (Figure 2A). We separated subcutaneous fat and peri-epidermal fat at the time of sampling and weighed them. Fat mass did not differ significantly under the NCD and HFD states (Figure 2B,C). Fasting blood glucose was reduced in KO mice under NCD; however, this reduction trend was no longer evident after HFD induction (Figure 2D). Glucose tolerance tests (GTT) demonstrated that KO mice showed impaired glucose tolerance, and this impairment was more severe after the HFD challenge (Figure 2E,F). The random blood glucose levels of KO and WT mice were not significantly different, regardless of the dietary condition (Figure 2G). To further explore the sensitivity of the body to insulin, insulin tolerance tests (ITT) were performed. The ITT results were not significantly different between the WT and KO groups (Figure 2H,I). Furthermore, we measured insulin levels upon glucose stimulation, and there was still no significant difference (Figure 2J). In addition, serum triglyceride (TG) and total cholesterol (TC) levels were not significantly altered (Figure 2K,L).

There was a slight increase in body weight in HFD-induced KO mice, but there was no significant difference in food intake (Figure S2A,B). Next, we evaluated the behavioral activity and energy homeostasis in WT and KO mice. WT and KO mice showed no difference in physical activity (Figure S2C). Indirect calorimetry showed that oxygen consumption (VO_2_), CO_2_ production (VCO_2_), and heat production did not change significantly (Figure S2D–F). However, the respiratory exchange ratio (RER) of KO mice was significantly lower than that of WT mice both during the day and night (Figure 2M,N). The lower RER may indicate that fatty acids are preferentially consumed for energy production compared to glucose. Taken together, the loss of the miR-23b/27b/24-1 cluster reduced the utilization of glucose, while insulin sensitivity and secretion were not affected.

### 2.3. miR-23b/27b/24-1 Cluster Knockout Alters the Glycolysis Pathway

**Figure 3:**
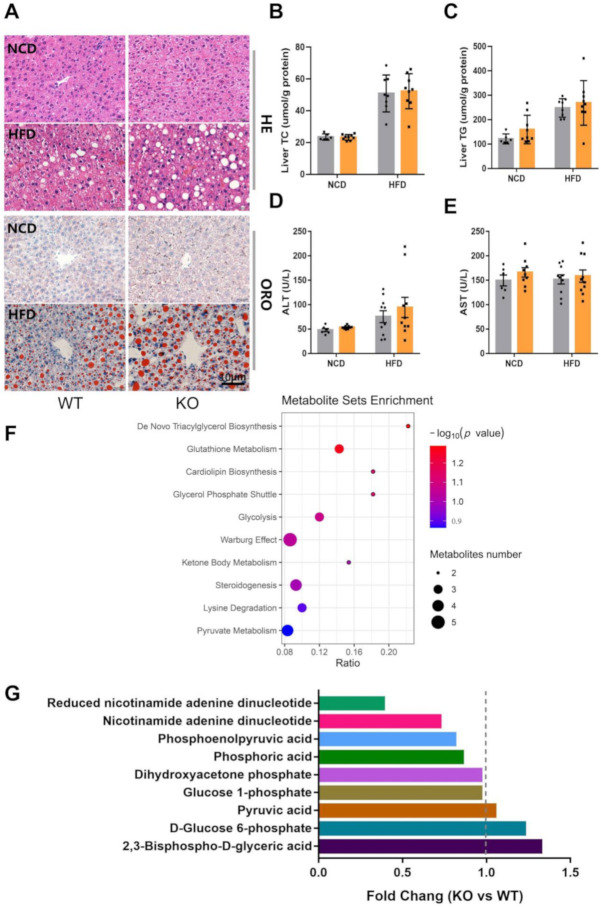
Ablation of miR-23b/27b/24-1 cluster alters glycolysis. (**A**) Representative images of hematoxylin and eosin (H&E)- and Oil-red O (ORO) -stained sections of liver from NCD or HFD-induced WT and KO mice. Scale bar: 10 μm. (**B**) Levels of hepatic cholesterol and triglycerides (**C**) of WT and KO mice fed an NCD (*n* = 6–9) or HFD (*n* = 8–9). (**D**) Levels of alanine aminotransferase (ALT) and (**E**) aspartate aminotransferase (AST) of WT and KO mice fed an NCD (*n* = 6–9) or HFD (*n* = 10). (**F**) Metabolite enrichment analysis revealed the top 10 pathways that were enriched. (**G**) Expression of metabolites involved in glycolysis pathways.

Glucose tolerance was impaired after the ablation of the miR-23b/27b/24-1 cluster, but there was no significant difference in ITT and glucose-stimulated insulin secretion (GSIS). Therefore, we focused on the regulatory changes in the liver. Hepatic histological results showed that KO mice had slightly higher lipid accumulation than WT mice (Figure 3A), while total liver cholesterol was not significantly different (Figure 3B), and liver triglycerides tended to be slightly elevated (Figure 3C). The overall effect of knockout of miR-23b cluster on alanine aminotransferase (ALT) and aspartate aminotransferase (AST) was not significant (Figure 3D,E), which implies that there is little effect on liver function. To further explore the effects of the miR-23b/27b/24-1 cluster on the liver, we performed liver metabolomics assays. A total of 959 differential metabolites were identified by non-targeted metabolomics (Table S2). The enrichment analysis of the differential metabolites (*p* < 0.1) showed that glycolysis was highly enriched (Figure 3F). Then, we analyzed the expression of all metabolites involved in glycolysis. Although the level of terminal product pyruvate was not significantly altered, the levels of reduced nicotinamide adenine dinucleotide (NADH), nicotinamide adenine dinucleotide (NAD), phosphoric acid, and the intermediate product phosphoenolpyruvic acid (PEP), which are involved in glycolysis, were decreased (Figure 3G). These results give us a hint that the miR-23b/27b/24-1 cluster may be involved in the regulation of glycolysis.

### 2.4. MiR-23b/27b/24-1 Cluster Deletion Decreases Pyruvate Kinase

**Figure 4:**
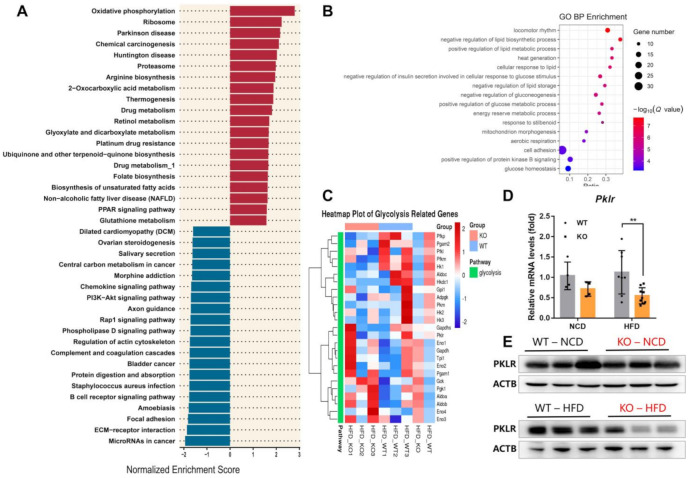
Deletion of miR-23b/27b/24-1 cluster decreases pyruvate kinase. (**A**) Top 20 dysregulated gene sets of RNA-seq data. (**B**) Gene Ontology (GO) enrichments analysis of dysregulated expressed genes (*q*-value < 0.001). (**C**) Heatmap of glycolysis-related genes. (**D**) qPCR of *Pklr* between WT and KO mice fed an NCD (*n* = 4) or an HFD (*n* = 8–9). (**E**) Protein expression of PKLR with miR-23b/27b/24-1 cluster ablation in the liver (*n* = 3). Data were represented as mean ± SD. Significance was determined by Student’s *t*-test analysis, ** *p* < 0.01.

To explore the specific alterations after miR-23b/27b/24-1 cluster ablation, we also performed an RNA-sequencing analysis of the liver. The threshold for differentially expressed genes was set at *q*-value <0.05. A total of 142 genes with >2-fold upregulation and 409 genes with >2-fold downregulation were identified (Figure S3 and Table S3). We randomly validated five genes with relatively high fold changes, high expression, and low *p*-values by qPCR, and the results were highly consistent with the RNA-seq data (Figure S4A,B). This indicates that the reliability of our RNA-seq results is robust. Gene set enrichment analysis (GSEA) was performed to investigate the enriched transcriptome changes due to ablation of the miR23b cluster. GSEA was based on the pathway database, which revealed 17 gene sets that were significantly upregulated and 15 gene sets that were downregulated in the KO mice compared to their WT littermates (*q*-value <0.05). The top 20 dysregulated gene sets were presented, including oxidative phosphorylation, non-alcoholic fatty liver disease (NAFLD), and the PI3K-Akt signaling pathway (Figure 4A). The top-ranked enrichment analysis terms of dysregulated expressed genes were a lipid metabolic process and glucose process (Figure 4B). We observed miR-23b/27b/24-1 cluster knockout mice exhibiting impaired glucose tolerance, in addition, liver metabolite enrichment analysis also directed to glycolysis. Then, we reviewed the expression of all genes involved in the glycolysis pathway (Figure 4C and Table S4). The heatmap indicated that genes involved in glycolysis showed a certain downregulation trend. *Pklr* encodes pyruvate kinase, which catalyzes PEP to pyruvate and was slightly reduced in the transcriptomic results. This reduction was more pronounced after extended sample validation, especially after the HFD induction (Figure 4D,E). Together with the reduced PEP, glycolysis was impaired with the deletion of the miR-23b/27b/24-1 cluster.

## 3. Discussion

In the present study, we investigated the effects of the miR-23b/27b/24-1 cluster on glucolipid metabolism in mice. The results showed that knockout of the miR-23b/27b/24-1 cluster in vivo impaired glucose tolerance. Metabolic cage experiments also showed that KO mice had a reduced RER, which coincided with impaired glucose utilization capacity. Subsequent multi-omics combination analysis showed that the miR-23b/27b/24-1 cluster is involved in the regulation of glycolysis in liver.

The regulation of glucose metabolism is complex and is not only dependent on hepatic controls but is also governed by peripheral tissues such as adipose, skeletal muscle, and the pancreas [27,28]. Our results showed that glucose tolerance was impaired after ablation of the miR-23b/27b/24-1 cluster, but there was no significant difference in ITT. Meanwhile, no difference was detected in the GSIS result. Therefore, we focused on the regulatory changes in the liver. The liver is crucial for maintaining normal glucose homeostasis, and hepatic glucose metabolism involves multiple catabolic and anabolic pathways [27]. Glucose is metabolized in the cytoplasm by the glycolytic pathway to pyruvate, while gluconeogenesis is a reversible reaction in glycolysis starting with pyruvate [29]. Under HFD, mice have impaired glucose tolerance and insulin resistance but unaltered levels of the gluconeogenic enzymes G6PC and PCK1 [30,31,32]. Moreover, reduced glycolysis during high-fat feeding suggests that there is a competition between fatty acids and glucose [33,34].

Our results showed a lower RER in KO mice than WT mice, indicating that fatty acids are preferentially consumed for energy production compared to glucose. Glycolysis is an important energy-producing process in mammalian cellular metabolism in which glucose is catalyzed to pyruvate, resulting in a net gain of two ATP and two NADH molecules from one glucose molecule [35]. Metabolomics showed that with miR-23b/27b/24-1 cluster ablation, NADH in KO mice was less than half of that in WT mice. At the same time, RNA-seq analysis showed that most of the genes in the glycolysis pathway were downregulated. Pyruvate kinase (encoded by *Pklr*) plays an important role in catalyzing phosphoenolpyruvate to pyruvate [36]. Our results showed that PKLR was significantly reduced in knockout mice, both at the mRNA and protein levels. As expected, our non-targeted metabolomic results only revealed a slight reduction in phosphoenolpyruvate, while pyruvate did not change significantly. However, this should be clarified through further refined experimental methods such as targeted metabolomics/metabolic flow. Growing evidence has shown that key enzymes in the glycolytic pathway are affected by miRNAs and post-translational modifications (PTMs) [37,38,39]. In breast cancer, miR-27b has been reported to target the 3′ untranslated region (3′UTR) region of pyruvate dehydrogenase complex component X (*PDHX*), limiting the conversion of pyruvate in the tricarboxylic acid cycle (TCA) [40]. It has also been shown that miR-24 can target lactate dehydrogenase A (*LDHA*) and restrict the conversion of pyruvate to lactate [41]. To our knowledge, this is the first report of three miRNAs, miR-23b, miR-27b, and miR-24, whose simultaneous knockout affects glycolysis. The specific regulatory mechanism remains to be further investigated.

Overall, our results demonstrated that loss of the miR-23b/27b/24-1 cluster impairs glucose tolerance via the glycolysis pathway.

## 4. Materials and Methods

### 4.1. Animals

Mice were housed in an environment with stable temperature (20–22 °C), 12/12 h light/dark cycle, 50–70% humidity, and provided with adequate food and water at periodic intervals. Ob/ob and ob/+ male mice tissues were kindly gifted by Dr. Wanzhu Jin (Key Laboratory of Animal Ecology and Conservation Biology, Institute of Zoology, Chinese Academy of Sciences, Beijing, China). MiR-23b/27b/24-1 cluster heterozygous mice were obtained using Clustered Regularly Interspersed Short Palindromic Repeats (CRISPR)-Cas9 technology. Knockout (KO) mice were generated by crossbreeding between heterozygous mice. Littermate wild-type (WT) mice were used as controls. Six-week-old mice were fed with a normal chow diet (NCD, D12450B; Research Diets, New Brunswick, NJ, USA) or a high-fat diet (HFD, D12492; Research Diets, New Brunswick, NJ, USA) consisting of 60% fat. Body weight was monitored weekly, and food intake was monitored for 10 consecutive days at 8 weeks of different dietary induction. The experiments were conducted 12–14 weeks after different dietary inductions. Afterwards, the mice were euthanized after a 6 h fast for sample collection. All experimental mice were male mice with a C57BL/6 background. Genotyping was performed by PCR using DNA isolated from the tail, and the primers are shown in Table S1. All animal research protocol was approved by the Animal Care and Use Committee of Center for Reproductive Medicine, Shandong University (2018-0017).

### 4.2. Metabolic Studies

Glucose tolerance tests (GTTs) were performed after 16 h of overnight fasting. KO and WT mice under different dietary conditions were injected intraperitoneally (i.p.) with glucose (2 g/kg for NCD and 1.5 g/kg for HFD). Blood glucose concentrations were measured at 0, 15, 30, 60, and 120 min after injection. For the insulin tolerance test (ITT), mice were fasted for 6 h before receiving an i.p. injection of insulin (0.75 U/kg for NCD and 1.0 U/kg for HFD). Blood glucose concentrations were measured at 0, 15, 30, 45, and 60 min after injection. The total area under the curve (AUC) was calculated using the trapezoid rule. For glucose-stimulated insulin secretion (GSIS) assay, mice were fasted for 16 h before receiving an i.p. injection of glucose. Blood was collected via the tail vein before and 30 min after injection, and the serum was separated and stored at −80 °C until insulin measurement.

### 4.3. Metabolic Cage

To analyze the metabolic status of the KO mice, metabolic cages (Columbus Instruments, OH, USA) were used to detect oxygen consumption (VO_2_), CO_2_ production (VCO_2_), respiratory exchange ratio (RER), heat production, and total activity (XTOT). Experiments involving WT and KO mice were performed after 14 weeks of HFD challenge (*n* = 4 in each group). Mice were individually monitored for three successive days, and data were collected on the last two days after 1-day adaptation.

### 4.4. Liver Histological Analysis

Livers were isolated at a given time and fixed in 4% formaldehyde for 24 h. Then, fixed tissues were dehydrated and processed for paraffin embedding, and 5-μm sections were stained with hematoxylin and eosin (H&E). For Oil Red O (ORO) staining, specific-sized liver tissues were embedded in optimal cutting temperature compound (OCT) medium and snap-frozen in liquid nitrogen. Then, 10-μm sections were stained with Oil Red O (Sigma–Aldrich, MO, USA). The slides were photographed under an inverted microscope (Olympus, Tokyo, Japan).

### 4.5. Insulin and Lipid Profile Measurement

Serum insulin levels at different time points were measured by a sandwich ELISA with a Rat/Mouse Insulin ELISA kit (Millipore, Billerica, MA, USA). Serum ALT and AST were measured at the Northern Institute of Biotechnology in Beijing. Total cholesterol and triglycerides in serum were measured using commercial kits (Applygen Technologies, Inc., Beijing, China). After liver tissue homogenization, total cholesterol and triglycerides were measured using commercial kits (Applygen Technologies, Inc., Beijing China) and normalized by total liver protein. Therefore, all operations were performed according to the kit instructions and finally measured on a multifunctional plate reader (Molecular Devices, San Jose, CA, USA).

### 4.6. Western Blotting

To prepare protein extracts, livers were harvested from WT and KO mice, and total protein was extracted using a commercial Minute™ Total Protein Extraction Kit (Invent, Beijing, China) plus protease inhibitors (Beyotime, Shanghai, China). Protein concentrations were measured using the BCA method (Thermo Fisher, Waltham, MA, USA). Equal amounts of protein were electrophoresed on 4–12% Bis-Tris gels, and the protein bands were transferred to polyvinylidene fluoride membranes (Millipore, MA, USA). The primary antibodies for immunoblotting included PKLR (Proteintech Group, Wuhan, Hubei, China) and β-actin (Sigma-Aldrich, St Louis, MO, USA). Immunoreactive bands were detected using Immobilon Western HRP Substrate Peroxide Solution (Millipore, Billerica, MA, USA) and analyzed with a Bio-Rad ChemiDoc MP Imaging System and Image Lab Software (Bio-Rad, Hercules, CA, USA).

### 4.7. mRNA and miRNA qRT-PCR

Total RNA was extracted using the Tissue RNA Purification Kit Plus according to the manufacturer’s instructions (ES Science, Shanghai, China). For mRNA analysis, total RNA was reverse transcribed to cDNA using the Prime Script RT Kit with gDNA Eraser (Takara, Shiga, Japan). Hairpin-it^TM^ miRNAs qPCR quantitation assay was performed to detect the expression of mature miRNAs s (GenePharma, Shanghai, China). qRT-PCR was performed using the SYBR Premix Ex Taq kit (Takara, Shiga, Japan) on a Light Cycler 480 System (Roche, Basel, Switzerland). The primer pairs used are presented in Table S1. Gene expression results were normalized with *β-actin, Gapdh*, and *U6* as housekeeping genes. The relative quantification values were calculated using the 2^−ΔΔCt^ method.

### 4.8. RNA Sequencing

Total RNA was extracted from WT and KO livers under the HFD challenge (*n* = 3). Transcriptome sequencing experiments were performed by BGI·Tech Company (Wuhan, China). The transcriptome library was generated after analyzing the concentration and RNA integrity number using an Agilent 2100 Bioanalyzer (Agilent Technologies, Santa Clara, CA, USA). After fragment screening, library building, and PCR product purification, the samples were sequenced on the BGISEQ-500 platform (Wuhan, China). The differentially expressed genes were identified with a *q*-value <0.05 and a fold-change >2 between the two groups.

### 4.9. Untargeted Metabolomics

Livers were individually ground with liquid nitrogen, and the homogenate was resuspended with prechilled 80% methanol and 0.1% formic acid and then mixed well by vortex (*n* = 4). The samples were incubated on ice for 5 min and then centrifuged at 15,000× rpm at 4 °C for 5 min. After another centrifugation at 15,000 rpm at 4 °C for 10 min, the supernatant was injected into the LC-MS/MS system analysis. UHPLC-MS/MS analyses were performed using a Vanquish UHPLC system (Thermo Fisher, Waltham, MA, USA) coupled with an Orbitrap Q ExactiveTM HF-X mass spectrometer (Thermo Fisher, Waltham, MA, USA) in Novogene Co., Ltd. (Beijing, China). The raw data files generated by UHPLC-MS/MS were processed using Compound Discoverer 3.1 (Thermo Fisher, Waltham, MA, USA) to perform peak alignment, peak picking, and quantitation for each metabolite. Metabolites with Variable Importance in Projection (VIP) > 1 and *p*-value <0.05 were considered as differential metabolites.

### 4.10. Data Analysis

MetaboAnalyst 4.0 (https://www.metaboanalyst.ca/) was applied for metabolite set enrichment analysis [42]. The global pathway enrichment analysis of RNA seq data was performed using an online website (http://www.webgestalt.org/#). The enrichment plots were generated with R (v.3.6.1, Auckland, NZ) ggplot package. A heatmap of glycolysis-related genes was plotted using Hiplot (https://hiplot.com.cn/). Histograms and line graphs were drawn using GraphPad Prism 7 software (GraphPad Software, San Diego, CA, USA). Data were expressed as the mean ± SD of at least three independent experiments. Statistical analysis was performed using the two-tailed Student’s *t*-test, and statistical significance was considered at *p* < 0.05.

## Data Availability

The data presented in this study are available on request from the corresponding author.

## Supplementary Materials

The following are available online at https://www.mdpi.com/1422-0067/22/2/550/s1, Figure S1: Expression pattern of the miR-23b cluster, Figure S2: Overall metabolic alterations in KO mice, Figure S3: Liver transcriptome profiles after ablation of miR-23b cluster, Figure S4: Validation of RNA-seq results, Table S1: Primer sequences for quantitative real-time PCR, Table S2: Differential metabolites identified by non-targeted metabolomics Table S3: All differentially expressed genes, Table S4: Genes involved in the glycolysis pathway.

## References

[1] Tancredi M., Rosengren A., Svensson A.M., Kosiborod M., Pivodic A., Gudbjörnsdottir S., Wedel H., Clements M., Dahlqvist S., Lind M. (2015). Excess mortality among persons with type 2 diabetes. N. Engl. J. Med..

[2] Cowie C.C. (2019). Diabetes diagnosis and control: Missed opportunities to improve health: The 2018 Kelly West Award Lecture. Diabetes Care.

[3] Heneghan H.M., Miller N., Kerin M.J. (2010). Role of microRNAs in obesity and the metabolic syndrome. Obes. Rev..

[4] Fernandez-Valverde S.L., Taft R.J., Mattick J.S. (2011). MicroRNAs in β-cell biology, insulin resistance, diabetes and its complications. Diabetes.

[5] Garavelli S., Bruzzaniti S., Tagliabue E., di Silvestre D., Prattichizzo F., Mozzillo E., Fattorusso V., La Sala L., Ceriello A., Puca A.A. (2020). Plasma circulating miR-23~27~24 clusters correlate with the immunometabolic derangement and predict C-peptide loss in children with type 1 diabetes. Diabetologia.

[6] McAlinden A., Varghese N., Wirthlin L., Chang L.W. (2013). Differentially expressed microRNAs in chondrocytes from distinct regions of developing human cartilage. PLoS ONE.

[7] He H.C., Zhu J.G., Chen X.B., Chen S.M., Han Z.D., Dai Q.S., Ling X.H., Fu X., Lin Z.Y., Deng Y.H. (2012). MicroRNA-23b downregulates peroxiredoxin III in human prostate cancer. FEBS Lett..

[8] Bang C., Fiedler J., Thum T. (2012). Cardiovascular importance of the microRNA-23/27/24 family. Microcirculation.

[9] Ouda R., Onomoto K., Takahasi K., Edwards M.R., Kato H., Yoneyama M., Fujita T. (2011). Retinoic acid-inducible gene I-inducible miR-23b inhibits infections by minor group rhinoviruses through down-regulation of the very low density lipoprotein receptor. J. Biol. Chem..

[10] Zhu S., Pan W., Song X., Liu Y., Shao X., Tang Y., Liang D., He D., Wang H., Liu W. (2012). The microRNA miR-23b suppresses IL-17-associated autoimmune inflammation by targeting TAB2, TAB3 and IKK-α. Nat. Med..

[11] Brovkina O., Nikitin A., Khodyrev D., Shestakova E., Sklyanik I., Panevina A., Stafeev I., Menshikov M., Kobelyatskaya A., Yurasov A. (2019). Role of MicroRNAs in the regulation of subcutaneous white adipose tissue in individuals with obesity and without type 2 diabetes. Front. Endocrinol..

[12] Henriksen T.I., Davidsen P.K., Pedersen M., Schultz H.S., Hansen N.S., Larsen T.J., Vaag A., Pedersen B.K., Nielsen S., Scheele C. (2017). Dysregulation of a novel miR-23b/27b-p53 axis impairs muscle stem cell differentiation of humans with type 2 diabetes. Mol. Metab..

[13] Hu X., Tang J., Hu X., Bao P., Pan J., Chen Z., Xian J. (2018). MiR-27b impairs adipocyte differentiation of human adipose tissue-derived mesenchymal stem cells by targeting LPL. Cell. Physiol. Biochem..

[14] Karbiener M., Fischer C., Nowitsch S., Opriessnig P., Papak C., Ailhaud G., Dani C., Amri E.Z., Scheideler M. (2009). microRNA miR-27b impairs human adipocyte differentiation and targets PPARgamma. Biochem. Biophys. Res. Commun..

[15] Prabu P., Rome S., Sathishkumar C., Gastebois C., Meugnier E., Mohan V., Balasubramanyam M. (2019). MicroRNAs from urinary extracellular vesicles are non-invasive early biomarkers of diabetic nephropathy in type 2 diabetes patients with the ‘Asian Indian phenotype’. Diabetes Metab..

[16] Wang X., Sundquist J., Zöller B., Memon A.A., Palmér K., Sundquist K., Bennet L. (2014). Determination of 14 circulating microRNAs in Swedes and Iraqis with and without diabetes mellitus type 2. PLoS ONE.

[17] Seyhan A.A., Nunez Lopez Y.O., Xie H., Yi F., Mathews C., Pasarica M., Pratley R.E. (2016). Pancreas-enriched miRNAs are altered in the circulation of subjects with diabetes: A pilot cross-sectional study. Sci. Rep..

[18] Nunez Lopez Y.O., Garufi G., Seyhan A.A. (2016). Altered levels of circulating cytokines and microRNAs in lean and obese individuals with prediabetes and type 2 diabetes. Mol. Biosyst..

[19] Demirsoy İ.H., Ertural D.Y., Balci Ş., Çınkır Ü., Sezer K., Tamer L., Aras N. (2018). Profiles of circulating MiRNAs following metformin treatment in patients with type 2 diabetes. J. Med. Biochem..

[20] Alicka M., Major P., Wysocki M., Marycz K. (2019). Adipose-derived mesenchymal stem cells isolated from patients with type 2 diabetes show reduced “stemness” through an altered secretome profile, impaired anti-oxidative protection, and mitochondrial dynamics deterioration. J. Clin. Med..

[21] Kokkinopoulou I., Maratou E., Mitrou P., Boutati E., Sideris D.C., Fragoulis E.G., Christodoulou M.I. (2019). Decreased expression of microRNAs targeting type-2 diabetes susceptibility genes in peripheral blood of patients and predisposed individuals. Endocrine.

[22] Avgeris M., Kokkinopoulou I., Maratou E., Mitrou P., Boutati E., Scorilas A., Fragoulis E.G., Christodoulou M.I. (2020). Blood-based analysis of 84 microRNAs identifies molecules deregulated in individuals with type-2 diabetes, risk factors for the disease or metabolic syndrome. Diabetes Res. Clin. Pract..

[23] Vickers K.C., Shoucri B.M., Levin M.G., Wu H., Pearson D.S., Osei-Hwedieh D., Collins F.S., Remaley A.T., Sethupathy P. (2013). MicroRNA-27b is a regulatory hub in lipid metabolism and is altered in dyslipidemia. Hepatology.

[24] Ng R., Wu H., Xiao H., Chen X., Willenbring H., Steer C.J., Song G. (2014). Inhibition of microRNA-24 expression in liver prevents hepatic lipid accumulation and hyperlipidemia. Hepatology.

[25] Kurkewich J.L., Boucher A., Klopfenstein N., Baskar R., Kapur R., Dahl R. (2018). The mirn23a and mirn23b microrna clusters are necessary for proper hematopoietic progenitor cell production and differentiation. Exp. Hematol..

[26] Oikawa S., Wada S., Lee M., Maeda S., Akimoto T. (2018). Role of endothelial microRNA-23 clusters in angiogenesis in vivo. Am. J. Physiol. Heart Circ. Physiol..

[27] Petersen M.C., Vatner D.F., Shulman G.I. (2017). Regulation of hepatic glucose metabolism in health and disease. Nat. Rev. Endocrinol..

[28] Goodpaster B.H., Sparks L.M. (2017). Metabolic flexibility in health and disease. Cell Metab..

[29] Hers H.G., Hue L. (1983). Gluconeogenesis and related aspects of glycolysis. Annu. Rev. Biochem..

[30] Perry R.J., Zhang D., Zhang X.M., Boyer J.L., Shulman G.I. (2015). Controlled-release mitochondrial protonophore reverses diabetes and steatohepatitis in rats. Science.

[31] Perry R.J., Camporez J.G., Kursawe R., Titchenell P.M., Zhang D., Perry C.J., Jurczak M.J., Abudukadier A., Han M.S., Zhang X.M. (2015). Hepatic acetyl CoA links adipose tissue inflammation to hepatic insulin resistance and type 2 diabetes. Cell.

[32] Chen M., Wu L., Wu F., Wittert G.A., Norman R.J., Robker R.L., Heilbronn L.K. (2014). Impaired glucose metabolism in response to high fat diet in female mice conceived by in vitro fertilization (IVF) or ovarian stimulation alone. PLoS ONE.

[33] Schmid G.M., Converset V., Walter N., Sennitt M.V., Leung K.Y., Byers H., Ward M., Hochstrasser D.F., Cawthorne M.A., Sanchez J.C. (2004). Effect of high-fat diet on the expression of proteins in muscle, adipose tissues, and liver of C57BL/6 mice. Proteomics.

[34] Patel D.P., Krausz K.W., Xie C., Beyoğlu D., Gonzalez F.J., Idle J.R. (2017). Metabolic profiling by gas chromatography-mass spectrometry of energy metabolism in high-fat diet-fed obese mice. PLoS ONE.

[35] Li J., Wang T., Xia J., Yao W., Huang F. (2019). Enzymatic and nonenzymatic protein acetylations control glycolysis process in liver diseases. FASEB J..

[36] Yang W., Lu Z. (2015). Pyruvate kinase M2 at a glance. J. Cell. Sci..

[37] Bond S.T., Howlett K.F., Kowalski G.M., Mason S., Connor T., Cooper A., Streltsov V., Bruce C.R., Walder K.R., McGee S.L. (2017). Lysine post-translational modification of glyceraldehyde-3-phosphate dehydrogenase regulates hepatic and systemic metabolism. FASEB J..

[38] Xia M., Feng S., Chen Z., Wen G., Zu X., Zhong J. (2020). Non-coding RNAs: Key regulators of aerobic glycolysis in breast cancer. Life Sci..

[39] Hitosugi T., Chen J. (2014). Post-translational modifications and the Warburg effect. Oncogene.

[40] Eastlack S.C., Dong S., Ivan C., Alahari S.K. (2018). Suppression of PDHX by microRNA-27b deregulates cell metabolism and promotes growth in breast cancer. Mol. Cancer.

[41] Saumet A., Vetter G., Bouttier M., Antoine E., Roubert C., Orsetti B., Theillet C., Lecellier C.H. (2012). Estrogen and retinoic acid antagonistically regulate several microRNA genes to control aerobic glycolysis in breast cancer cells. Mol. Biosyst..

[42] Chong J., Wishart D.S., Xia J. (2019). Using MetaboAnalyst 4.0 for comprehensive and integrative metabolomics data analysis. Curr. Protoc. Bioinform..

