# Supplementary material for: Loss of miR-23b/27b/24-1 Cluster Impairs Glucose Tolerance via Glycolysis Pathway in Mice
Source: Int J Mol Sci. 2021 Jan 7;22(2):550. doi: 10.3390/ijms22020550 (PMC7826568; doi:10.3390/ijms22020550)
Supplement: Supplementary file 1 [file ijms-22-00550-s001.zip › ijms-1024855-suppl.-proof checked V2.pdf]

International Journal of
Molecular Sciences

MDPI

Supplementary Materials

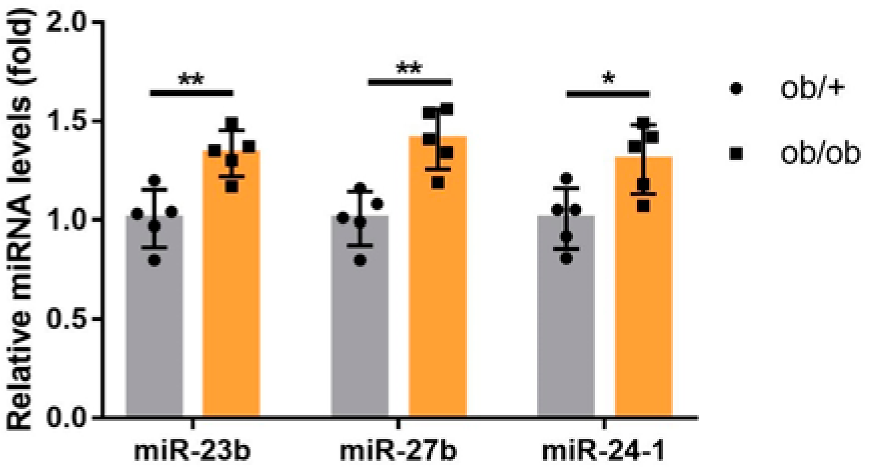

Figure S1. Expression pattern of the miR-23b cluster. miR-23b, miR-27b, and miR-24-1 were increased in the liver of ob/ob mice (n = 4–5). Data are represented as mean ± SEM. Significance was determined by Student's t-test analysis, * $p < 0.05$, ** $p < 0.01$.

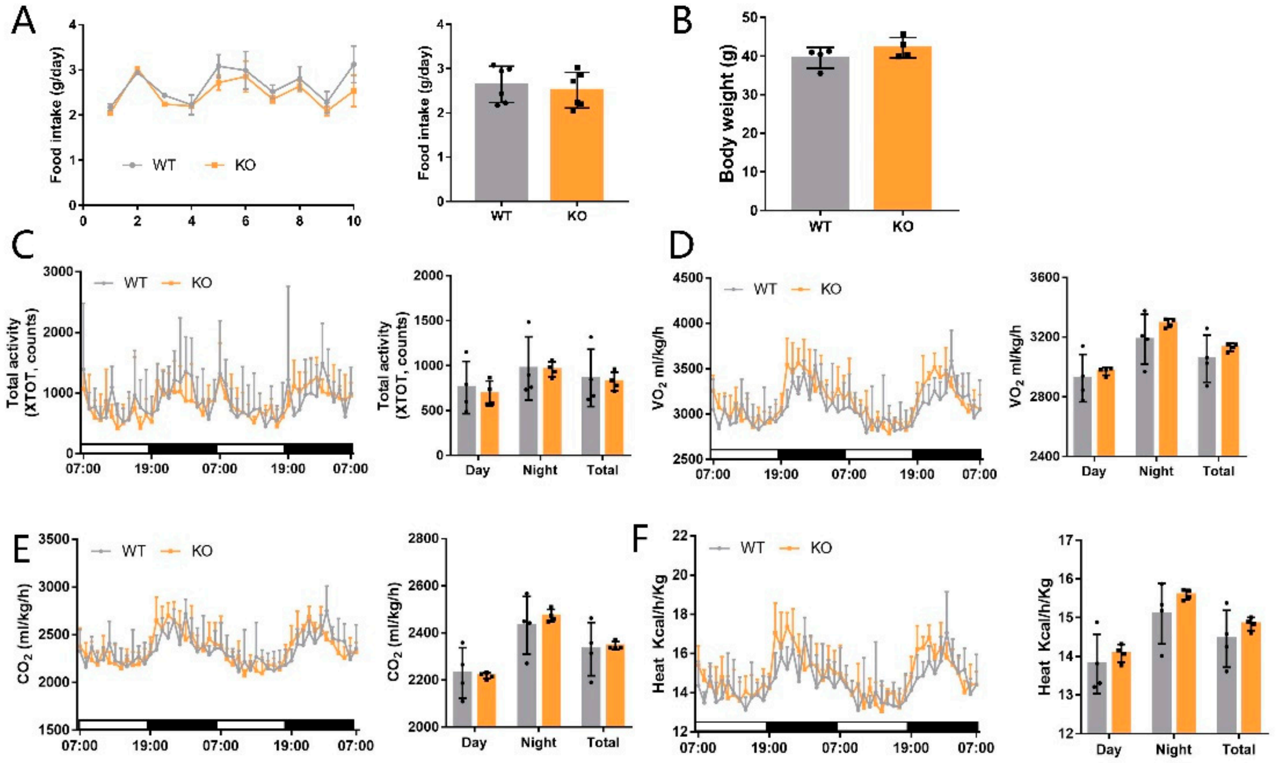

Figure 2. Overall metabolic alterations in knockout mice. (**A**) Continuous monitoring of food intake for 10 days in WT and KO mice fed an HFD (n = 6). Right panel, average food intake per mouse. (**B**) Body weights of KO and WT mice before metabolic cage experiments. (**C**–**F**) Total activity (XTOT), Oxygen consumption (VO_2), CO_2 production (VCO_2), and heat production were measured during 2 successive days using metabolic cages. The daytime and night-time averages are on the right panel. Mice were feed under HFD (n = 4v4). Data are represented as mean ± SD. Significance was determined by Student's t test analysis.

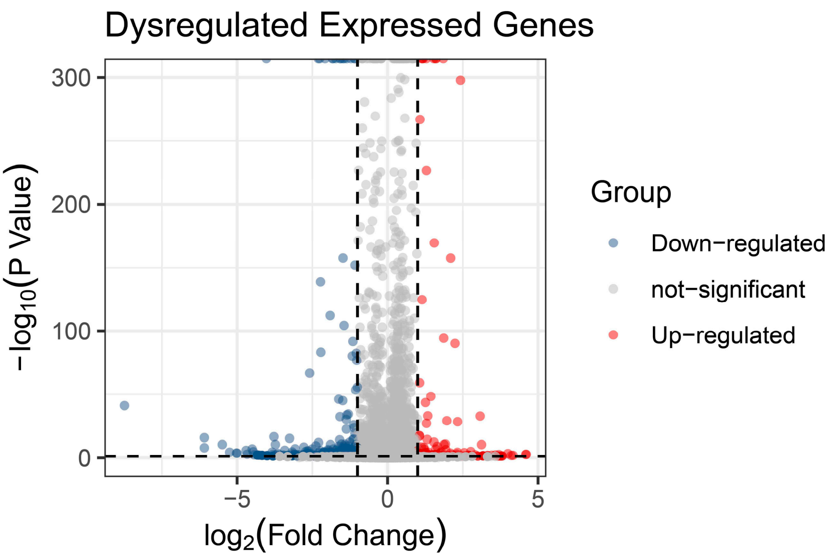

Figure S3. Liver transcriptome profiles after ablation of miR-23b cluster. Dysregulated expressed genes in miR23b cluster-deficient liver tissue compared with WT mice under HFD. The threshold is set to ∣Log2 FC∣ > 1 and Q-value <0.05. Red dots represent upregulated and blue dots represent downregulated.

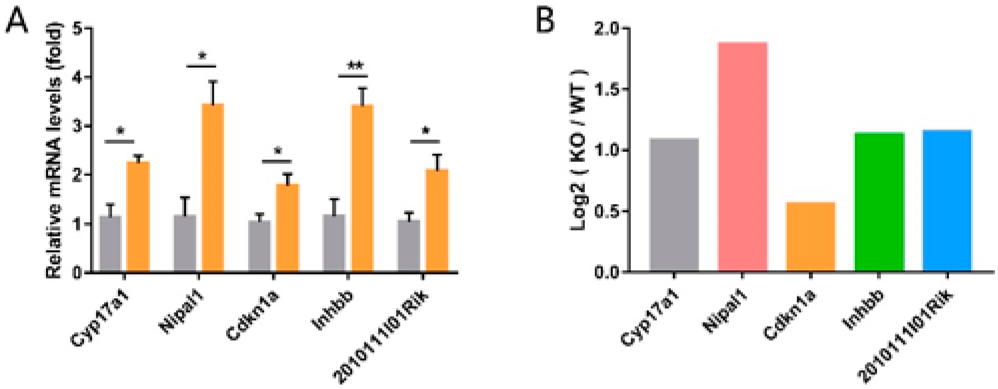

Figure S4. Validation of RNA-seq results. (**A**) qPCR analysis of mRNA levels from RNA-seq data with relatively high fold changes, high expression, and low *p*-values ($n = 4$). (**B**) Log2 FC of validated genes from transcriptomics. Data are represented as mean ± SEM. Significance was determined by Student's *t* test analysis, * $p < 0.05$, ** $p < 0.01$.

Table S1. Primer sequences for quantitative real-time PCR.

Target	Forward Primer (5'-3')	Reverse Primer (5'-3')
N209	GCTGTGTTGTCAAGAGTGCA	CAGCAGGGACAAAGAACCAC
T1348	CCAGAGACATCCCAACCCAT	AGACAGGCATTCTCACTGCT
Pklr	GATCCGAAGTTCCGGACAAGG	ATGAGCCCGTCGTCAATGTAG
Cyp17a1	GGCACTGCATCACGATAA	TCCGAAGGGCAAATAACT
Nipal1	GTCAGAGTCGCTGCCTTATCC	TGCAAGAGACCCTTCTTTTTGAG
Cdkn1a	CCTGGTGATGTCCGACCTG	CCATGAGCGCATCGCAATC
Inhbb	CTTCGTCTCTAATGAAGGCAACC	CTCCACCACATTCCACCTGTC
2010111I01Rik	GTCAACATGGCAGGCTACAG	GGGCTTCATTTCTGTCCAGC
β-actin	CATCCGTAAAGACCTCTATGCCAAC	ATGGAGCCACCGATCCACA
Gapdh	AGGTCGGTGTGAACGGATTTG	TGTAGACCATGTAGTTGAGGTCA
miR-23b-3p	GGCAGACAATCACATTGCCA	TATGGTTGTTCACGACTCCTTCAC
miR-27b-3p	CTCGTCAATTCACAGTGGCTAAG	TATGGTTTTGACGACTGTGTGAT
miR-24-3p	CCGGTGCCTACTGAGCTGATAT	TATCCTTCTTCACGACTCCTTCAC
U6	CAGCACATATACTAAAATTGGAACG	ACGAATTTGCGTGTCATCC
